# Supplementary material for: Orbital frontal cortex updates state-induced value change for decision-making
Source: eLife. 2018 Jun 13;7:e35988. doi: 10.7554/eLife.35988 (PMC6039177; doi:10.7554/eLife.35988)
Supplement: Supplementary file 1. [file elife-35988-supp1.docx]

**Supplementary Material**

Title: Orbital frontal cortex updates state-induced value change for decision-making

Authors and author addresses:

Emily T. Baltz^1^, Ege A. Yalcinbas^1,2^, Rafael Renteria^1^, and Christina M. Gremel, Ph.D.^1, 2*^.

^1^Department of Psychology, University of California San Diego, La Jolla, CA 92093, USA

^2^ The Neurosciences Graduate Program, University of California San Diego, La Jolla, CA 92093, USA

Supplementary Table 1: Effect of Strain on Responses

| **Group** | **stage** | **comparison** | **n** | **Stats (ANOVA)** | **F value** | **P value** |
| --- | --- | --- | --- | --- | --- | --- |
| **Positive Incentive Learning** | acquisition | response rate | 2-2 Ctl: 9  2-16 Ctl:14  2-2 OFC: 7  2-16 OFC: 17 | Food Restriction x Strain x Tx | F(1,39) = 0.48 | 0.49 |
|  |  |  |  | Food Restriction x Strain | F(1,39) = 0.31 | 0.58 |
|  |  |  |  | Food Restriction x Tx | F(1,39) = 0.12 | 0.74 |
|  |  |  |  | Strain x Tx | F(1,39) = 0.25 | 0.62 |
|  |  |  |  | Food Restriction | F(1,39) = 2.32 | 0.13 |
|  |  |  |  | Strain | F(1,39) = 0.96 | 0.33 |
|  |  |  |  | Tx | F(1,39) = 0.09 | 0.76 |
|  | re-exposure | licking rate | 2-2 Ctl: 9  2-16 Ctl:14  2-2 OFC: 7  2-16 OFC: 17 | Food Restriction x Strain x Tx | F(1,38) = 0.14 | 0.71 |
|  |  |  |  | Food Restriction x Strain | F(1,38) = 0.25 | 0.62 |
|  |  |  |  | Food Restriction x Tx | F(1,38) =2.37 | 0.13 |
|  |  |  |  | Strain x Tx | F(1,38) = 01.30 | 0.26 |
|  |  |  |  | **Food Restriction** | **F(1,38) = 25.78** | **<0.001** |
|  |  |  |  | Strain | F(1,38) = 0.41 | 0.53 |
|  |  |  |  | Tx | F(1,38) = 1.93 | 0.17 |
|  | test | % baseline left lever presses | 2-2 Ctl: 9  2-16 Ctl:14  2-2 OFC: 7  2-16 OFC: 17 | Food Restriction x Strain x Tx | F(1,39) < 0.01 | 0.98 |
|  |  |  |  | Food Restriction x Strain | F(1,39 )= 1.12 | 0.30 |
|  |  |  |  | Food Restriction x Tx | F(1,39) = 3.83 | 0.05 |
|  |  |  |  | Strain x Tx | F(1,39) = 0.56 | 0.46 |
|  |  |  |  | Food Restriction | F(1,39) = 1.13 | 0.29 |
|  |  |  |  | Strain | F(1,39) = 0.47 | 0.49 |
|  |  |  |  | Tx | F(1,39) = 0.93 | 0.34 |
|  | | | | | | |
| **Negative Incentive Learning** | acquisition | response rate | 16-16 Ctl: 19  16-2 Ctl: 16  16-16 OFC: 16  16-2 OFC: 17 | Food Restriction x Strain x Tx | F(1,60) = 0.35 | 0.55 |
|  |  |  |  | Food Restriction x Strain | F(1,60) = 0.48 | 0.49 |
|  |  |  |  | Food Restriction x Tx | F(1,60) = 0.21 | 0.65 |
|  |  |  |  | Strain x Tx | F(1,60) = 0.24 | 0.63 |
|  |  |  |  | Food Restriction | F(1,60) = .09 | 0.76 |
|  |  |  |  | Strain | F(1,60 )= 0.002 | 0.96 |
|  |  |  |  | Tx | F(1,60) = 1.58 | 0.21 |
|  | re-exposure | licking rate | 16-16 Ctl: 19  16-2 Ctl: 16  16-16 OFC: 16  16-2 OFC: 17 | Food Restriction x Strain x Tx | F(1,60) = 0.08 | 0.78 |
|  |  |  |  | Food Restriction x Strain | F(1,60) = 2.30 | 0.13 |
|  |  |  |  | Food Restriction x Tx | F(1,60) = 0.08 | 0.78 |
|  |  |  |  | Strain x Tx | F(1,60) = 0.002 | 0.96 |
|  |  |  |  | **Food Restriction** | **F(1,60) = 9.92** | **0.003** |
|  |  |  |  | Strain | F(1,60) = 0.99 | 0.32 |
|  |  |  |  | Tx | F(1,60) = 0.15 | 0.70 |
|  | test | % baseline left lever presses | 16-16 Ctl: 19  16-2 Ctl: 16  16-16 OFC: 16  16-2 OFC: 17 | Food Restriction x Strain x Tx | F(1,60) = 0.04 | 0.84 |
|  |  |  |  | Food Restriction x Strain | F(1,60) = 0.37 | 0.55 |
|  |  |  |  | Food Restriction x Tx | F(1,60) = 2.22 | 0.14 |
|  |  |  |  | Strain x Tx | F(1,60) = 0.15 | 0.70 |
|  |  |  |  | Food Restriction | F(1,60) = 0.89 | 0.35 |
|  |  |  |  | Strain | F(1,60) = 0.22 | 0.64 |
|  |  |  |  | Tx | F(1,60) = 1.22 | 0.27 |

Supplementary Table 2: Comparison of Saline vs. CNO Treated Controls

| **Group** | **Stage** | **comparison** | **Control ns** | **effect** | **statistics** | **P value** |
| --- | --- | --- | --- | --- | --- | --- |
| **Positive incentive learning** | re-exposure | ANOVA (DrugxFoodrestriction) | 2-2 Sal: 5  2-2 CNO: 4  2-16 Sal:12  2-16 CNO:2 | Drug | 0.412 | 0.539 |
|  |  |  |  | **Food restriction** | **13.593** | **0.002** |
|  |  |  |  | Interaction | 0.001 | 0.970 |
|  | test | ANOVA (DrugxFoodrestricition) | 2-2 Sal: 5  2-2 CNO: 4  2-16 Sal:12  2-16 CNO: 2 | Drug | 0.164 | 0.690 |
|  |  |  |  | Foodrestriction | 1.093 | 0.309 |
|  |  |  |  | Interaction | 2.475 | 0.132 |
|  | | | | | | |
| **Negative incentive learning** | re-exposure | ANOVA (DrugxFoodrestriction) | 16-2 Sal: 10  16-2 CNO: 6  16-16 Sal: 8  16-16 CNO: 10 | Drug | 0.14905 | 0.702 |
|  |  |  |  | **Food restriction** | **17.440** | **0.001** |
|  |  |  |  | interaction | 0.994 | 0.382 |
|  | test | ANOVA (DrugxFoodrestriction) | 16-2 Sal: 10  16-2 CNO: 6  16-16 Sal: 8  16-16 CNO: 10 | Drug | F = 3.197 | 0.084 |
|  |  |  |  | **Food restriction** | **F = 5.985** | **0.021** |
|  |  |  |  | interaction | F = 0.743 | 0.396 |
